# Prospective Analytical Case-Control Study of COVID-19 Positive Versus Negative Patients for Subclinical Placental Dysfunction

**DOI:** 10.30699/ijp.2024.2017566.3225

**Published:** 2024-07-24

**Authors:** Poojan Dogra Marwaha, Jyoti Bala, Suchi Sharma, Asmita Kaundal, Neha Chauhan

**Affiliations:** 1 Department of Obstetrics & Gynecology, All India Institute of Medical Sciences (AIIMS), Bilaspur, H.P. India; 2 Department of Pathology, SLBS, Nerchowk, Mandi, H.P., India; 3 Department of Obstetrics & Gynecology, SLBS, Nerchowk, Mandi, H.P., India

**Keywords:** COVID-19, Pathology, Placenta, Pregnancy

## Abstract

**Background & Objective:**

Placenta provides nutrition and protection from various infections and toxins to the baby while they are in the mother’s womb. The present study was done to see the effects of coronavirus infection on the placenta of infected mothers and the final pregnancy outcome.

**Methods:**

A total of 50 antenatal women (25 infected with coronavirus and 25 uninfected women) were enrolled and their placentae were examined for any significant histopathological changes. These changes were then correlated with the final pregnancy outcome.

**Results:**

A significant number of placentae from infected mothers demonstrated features of maternal (54%) and fetal malperfusion (76%). However, no significant adverse pregnancy outcome was noted.

**Conclusion:**

Increased rates of maternal malperfusion, and fetal malperfusion may be seen in placentae from infected women. However, these changes may not progress to any adverse fetal outcomes.

## Introduction

The placenta not only provides nutrition, blood supply, and oxygenation, but also protects the growing fetus from toxins, infections, and environmental insults (1-4). However certain viruses and toxins may cross the placenta and affect the fetus resulting in abortion, congenital malformations, growth-restricted babies, in-utero fetal demise, or stillbirths (5-10). Placental pathologies not only alter the course of a normal pregnancy but can also affect the long-term health of the baby through childhood and later life. Though Coronavirus infected millions of pregnant women across the globe, the data related to feto-maternal outcomes in COVID-19 pregnancies has been conflicting. Cases have been published to demonstrate the virus-induced insult to the placenta of infected mothers but whether these damages have caused adverse outcomes is not established (11-15). 

Hence the present study was planned to examine coronavirus infection-induced changes in the placentae and resulting pregnancy outcomes considering these changes. To determine whether COVID-19-positive patients had an increased incidence of placental injury compared to a cohort of COVID-negative patients. Association of the COVID-19-induced placental damage with the final pregnancy outcomes (both maternal and fetal).

## Material and Methods

The present study was conducted as a prospective analytical case-control study after obtaining ethical clearance from the institutional ethical committee. A total of 50 women with singleton pregnancy, (twenty-five coronavirus-infected and 25 uninfected women) were enrolled. Women with known obstetric or medical conditions were not included in the study—the placentae of study participants after delivery were fixed with 10% formalin. The sample was kept for 1 week before processing to eliminate any risk of transmission of the virus. For the final evaluation, eight sections were taken from each placenta: maternal side of the placenta (2 sections), fetal side of the placenta (2 sections), full thickness (2 sections), membranes (1 section), and umbilical cord (1 section). Hematoxylin and eosin staining was done. A senior pathologist examined and noted all the findings on the predesigned proforma as per “Amsterdam consensus criteria” for any significant finding (16). 

Statistical analysis: Analysis of data was done using IBM SPSS software 26.0 (Armonk, NY, USA). Frequency along with the percentage of the categorical variables were taken. A P-value less than 0.05 was considered statistically significant.

## Results

Twenty-five, SARS-CoV-2 infective women carrying singleton otherwise uncomplicated pregnancy were enrolled in the study. The same number of age and gestational age-matched uninfected women were enrolled as control. The final sample included fifty placentae. The mean age of study participants was 24 ± 3.1 years. Most of the women among the cases were primigravida (60% cases versus 48% controls) however among controls most of the women were multiparous (52%controls versus 40% cases). The majority delivered at a gestational age between 37-40 weeks in both groups (64% versus 88%). Only two women in the cases and one woman in the control group had delivery before 37 weeks. One COVID-19 patient was referred for induction of labor at 34 weeks for intrauterine fetal death. Among the infected pregnant cases, the majority (92%) were asymptomatic. Around 4% had symptoms of mild COVID-19 infection and required no treatment or only symptomatic treatment, while 4% needed treatment for moderate disease. None of the patients had severe symptoms. Most women in cases had a cesarean section (76% cases versus 24% controls) whereas those among controls had a normal vaginal birth (76% control versus 24% cases). Detection of COVID-19 to the delivery interval was 1-3 days in the majority of the cases (72%) (Table 1).

Neonatal outcome is depicted in Table 2. No statistically significant difference was seen concerning birth weight, APGAR score, admission to the NICU, and birth asphyxia among cases and controls. Among the cases two (8%) neonates were COVID positive at birth and required NICU admission. 

**Table 1:** Maternal demographic characteristics

		Cases (N=25)	Controls (N=25)	P-value
Age distribution	20-25 years	12(48%)	11(44%)	0.999
	26-30 years	9(36%)	10(40%)	
	>30 years	4(16%)	4(16%)	
Parity distribution	Primigravida	15(60%)	12(48%)	0.394
	Multigravida	10(40%)	13(52%)	
Gestational age	<37 weeks	2(8%)	1(4%)	0.289
	37-40 weeks	16(64%)	22(88%)	
	>40 weeks	7(28%)	3(12%)	
Mode of delivery	FTVD	6(24%)	19(76%)	0.0001
	LSCS	19(76%)	6(24%)	
	Fetal heart abnormality	4(16%)	2(8%)	
	Nonprogress of labour	4(16%)	1(4%)	
	Malpresentation	4 (16%)	2(8%)	
	Placenta Previa	3(12%)	0(0%)	
	Abruption	1(4%)	1(4%)	
	Previous two LSCS	1(4%)	0(0%)	
	Cephalopelvic disproportion	2(8%)	0(0%)	
RT-PCR positive to delivery interval	1-3 days	18(72%)	-	
	3-6 days	07(28%)	-	

**Table 2:** Neonatal outcomes

Variable		Cases (n=25)	Controls(n=25)	P-value
Birth weight	1500-2000 gm	3(12%)	1(4%)	0.609
	2000-2500 gm	1(4%)	2(8%)	0.999
	2500-3000gm	10(40%)	10(40%)	1.000
	3000-3500gm	11(44%)	12(48%)	0.776
	>3500gm	2(8%)	4(16%)	0.667
Neonatal outcome	Alive	24(96%)	25(100%)	0.999
	Macerated IUFD	1(4%)	0	0.999
	Fresh stillbirth	0	0	
	Admission to NICU	4(16%)	0	0.109
	Birth asphyxia	2(8%)	Nil	0.489
	COVID status of the baby	2(8%)	Nil	0.489

Table 3 depicts the placental weight and histopathological findings in the placentae of the enrolled study participants. Placental weights were comparable in both groups. On the histopathological examination signs of maternal mal-perfusion were present in a significant proportion of the placentae from cases as compared to the controls (54% versus 8%). Placental infarcts (24% versus 1%), distal villous hypoplasia (20% versus 4%), acute fibrinoid necrosis (8% versus 0%), intervillous thrombosis (7% versus 4%), increased syncytial knots (12% versus 4%) and increased intervillous fibrin (32% versus 4%) were demonstrated on the histopathological examination. Around 76% of the placentae among the cases demonstrated signs of fetal malperfusion as compared to 8% in the placentae of the controls. Intramural fibrin deposition (76% versus 8%), and intravascular fibrin thrombi (28% versus 4%) were seen in the placentae of the cases. Inflammatory changes were also present in a higher number of placentae from infected women when compared with the controls (24% versus 20%). There was no significant difference in the placental features between those who delivered before or after 37 weeks and those who delivered babies with weight lesser than the expected weight for gestation compared to those with birth weight appropriate to gestational age babies. The Placenta of the women who presented with in-utero fetal demise showed marked villous edema. e Placenta of the mothers who gave birth to infected babies also showed features similar to the other placenta. Vascular ectasia (8% versus 0%), villous agglutination (28% versus 4%), and chorangiosis (36% versus 0%) were other histopathological findings seen in the placentae of infected women when compared to the placenta of the non-infected women.

**Table 3:** Histopathological changes in the placenta

Variable	Cases(N=25)	Controls (N=25)	P-value
Placental weight			
300-400	3(12%)	1(4%)	0.609
400-500	1(4%)	2(8%)	0.999
500-600	11(44%)	12(48%)	0.776
Signs of “maternal malperfusion”	14(54%)	2(8%)	0.0001
Placental infarcts	6(24%)	1(4%)	0.098
Distal villous hypoplasia	5(20%)	1(4%)	0.189
Decidual Arteriopathy	7(28%)	1(4%)	0.048
Acute fibrinoid necrosis	2(8%)	0	0.489
Intervillous thrombosis	7(28%)	1(4%)	0.048
Accelerated Villous Maturation	8(32%)	1(4%)	0.023
Increased syncytial knots	3(12%)	1(4%)	0.609
Increased intervillous fibrin	8(32%)	1(4%)	0.023
Retroplacental hemorrhage	0 (0%)	0 (0%)	0
Signs of “fetal vascular mal-perfusion”	19 (76%)	2 (8%)	0.0001
Intramural fibrin deposition	19 (76%)	2(8%)	0.0001
Intravascular thrombi	7 (28%)	1(4%)	0.048
Avascular villi	0 (0%)	0(0%)	0
Stromal vessel obliteration	0 (0%)	0(0%)	0
“Villous-stromal vascular karyorrhexis”	0 (0%)	0(0%)	0
Inflammatory changes	6 (24%)	5(20%)	0.732
Villitis	5 (20%)	0	0.050
Chorioamniotis	1 (4%)	5 (20%)	0.189
Other			
Vascular ectasia	2(8%)	0	0.489
Villous agglutination	7(28%)	1(4%)	0.048
Chorangiosis	9 (36%)	0	0.001

## Discussion

How infections/diseases affect pregnant women and neonates has always been of great interest to obstetricians and pediatricians. Histopathologic examination of placental tissue provides much information regarding the effect of maternal and fetal perfusion related to the disease process (17-21). Transplacental transmission of COVID-19 remained in debate for a long time (22-26). Vivantie *et al.* for the first time described the transmission of coronavirus through the transplacental route (27). The authors described “diffuse perivillous fibrin deposition with infarcts” and “intervillositis” in infected women’s placentae. The baby is required to be delivered through category II cesarean section for fetal heart rate abnormalities. The baby was found positive for the virus on testing. This case report prompted us to conduct this study and to clarify whether there is a particular coronavirus-induced pattern of placental pathologies and does these pathological changes translate into adverse pregnancy outcomes. 

Gao L *et al.* in their study described features of maternal malperfusion in eight placentae from COVID-infected women (28). The authors found increased evidence of “syncytial knots”, “focal or massive perivillous fibrin depositions”, “central or peripheral placental infarct”, and “distal villous hypoplasia” in these placentae. However, no feature of fetal vascular malperfusion was seen. In another study by Facchetti F *et al.* out of fourteen cases studied by the authors, perivillous fibrin deposition was seen in many areas of the placenta received after delivery of a baby who developed pneumonia soon after birth and was found to be positive for coronavirus, indicating transplacental transmission of the virus (29). In our study, intervillous thrombosis, increased intervillous fibrin and villous agglutination were statistically significant findings of maternal malperfusion in the histopathology of the placenta of the infected women as compared to the uninfected women. Shanes ED *et al.* in their study examined 16 placentae from COVID-19-positive women and confirmed the presence of maternal and fetal malperfusion in most placentae with the absence of any feature of acute or chronic inflammation (30). However, all the babies tested negative for COVID-19 at birth. Hence the authors concluded that most of the placental changes were due to maternal infection and vertical transmission was rare. Giordano *et al.* examined five placentae from COVID-19-positive women infected during late pregnancy who were convalescent during delivery (31). The placentae showed the features of maternal and fetal malperfusion however, none of the babies were positive for COVID-19 and with no adverse maternal and fetal outcomes. A multinational case-based retrospective study was done in 12 countries by David A Schwartz *et al.* to examine the placentae of 68 COVID-19-infected women who had stillbirth/early neonatal deaths to establish an association between placental changes induced by COVID-19 (placentitis) to the stillbirth (32). Placentitis was present in 97% of placentae. The most common cells involved were syncytiotrophoblasts in all the examined Placenta (100%). Cytotrophoblast cells (12%), Hofbauer cells (5%), villous cells (5%), villous capillary endothelial cells (3%), and extravillous trophoblasts cells were the other cells seen to be involved. On autopsy of these fetuses’ the majority found no cause however features of intrauterine hypoxia were present in 5 cases out of 68. The virus was present in the body specimen of 25 fetuses. Authors reported 2 cases of confirmed and 39 cases of possible maternal-fetal transmission among stillbirths. 

In the present study features of maternal malperfusion were present in around 55% (14/25) of placentae of infected women as compared to uninfected women 8% (2/25). Placenta infarcts (6/25), distal villous hypoplasia (5/25), acute fibrinoid necrosis (2/25), intervillous thrombosis (7/25), increased syncytial knots (3/25) and increased intervillous fibrin (8/25) were present. Features of fetal malperfusion (19/25) such as intramural fibrin deposition (19/25) and intravascular fibrin thrombi (7/25) were also present in the COVID-19-positive patient’s placentae. However, only two babies were COVID-19-positive at birth. No significant difference was seen in the histopathological changes in the placentae from mothers of the infected babies when compared to the placentae from mothers of uninfected babies.

## Conclusion

The placenta is an enigmatic organ that protects the fetus from toxins, infections, and disease. Though SARS-CoV2-induced changes may be seen in the placentae of COVID-19-positive women, however, these changes may not affect the outcome of pregnancy.
